# Circulating Inflamma-miRs as Potential Biomarkers of Cognitive Impairment in Patients Affected by Alzheimer’s Disease

**DOI:** 10.3389/fnagi.2021.647015

**Published:** 2021-03-11

**Authors:** Angelica Giuliani, Simona Gaetani, Giulia Sorgentoni, Silvia Agarbati, Maristella Laggetta, Giulia Matacchione, Mirko Gobbi, Tommaso Rossi, Roberta Galeazzi, Gina Piccinini, Giuseppe Pelliccioni, Anna Rita Bonfigli, Antonio Domenico Procopio, Maria Cristina Albertini, Jacopo Sabbatinelli, Fabiola Olivieri, Francesca Fazioli

**Affiliations:** ^1^Department of Clinical and Molecular Sciences, Università Politecnica Delle Marche, Ancona, Italy; ^2^Neurology Department, IRCCS INRCA, Ancona, Italy; ^3^Clinical Laboratory and Molecular Diagnostics, IRCCS INRCA, Ancona, Italy; ^4^Scientific Direction, IRCCS INRCA, Ancona, Italy; ^5^Center of Clinical Pathology and Innovative Therapy, IRCCS INRCA, Ancona, Italy; ^6^Department of Biomolecular Sciences, University of Urbino Carlo Bo, Urbino, Italy

**Keywords:** Alzheimer’s disease, circulating microRNAs, biomarker, inflammation, inflamma-miRs

## Abstract

Alzheimer’s disease (AD), the most prevalent neurodegenerative disease in the growing population of elderly people, is still lacking minimally-invasive circulating biomarkers that could facilitate the diagnosis and the monitoring of disease progression. MicroRNAs (miRNAs) are emerging as tissue-specific and/or circulating biomarkers of several age-related diseases, but evidence on AD is still not conclusive. Since a systemic pro-inflammatory status was associated with an increased risk of AD development and progression, we focused our investigation on a subset of miRNAs modulating the inflammatory process, namely inflamma-miRNAs. The expression of inflamma-miR-17-5p, -21-5p, -126-3p, and -146a-5p was analyzed in plasma samples from 116 patients with AD compared with 41 age-matched healthy control (HC) subjects. MiR-17-5p, miR-21-5p, and miR-126-3p plasma levels were significantly increased in AD patients compared to HC. Importantly, a strong inverse relationship was observed between miR-21-5p and miR-126-3p, and the cognitive impairment, assessed by Mini-Mental State Examination (MMSE). Notably, miR-126-3p was able to discriminate between mild and severe cognitive impairment. Overall, our results reinforce the hypothesis that circulating inflamma-miRNAs could be assessed as minimally invasive tools associated with the development and progression of cognitive impairment in AD.

## Introduction

Alzheimer’s disease (AD) is the most prevalent neurodegenerative disease, accounting for up to 80% of dementias worldwide (Alzheimer’s Association, 2020). AD is clinically associated with progressive memory loss and impairment of cognitive, functional, and behavioral domains (Lane et al., 2018). AD is typically classified into two categories based on the age of onset, i.e., early-onset AD (<65 years) and late-onset AD (LOAD; >65 years; Van Cauwenberghe et al., 2016). Increasing evidence suggests that sporadic LOAD can be considered as a chronic age-related disease (ARD), sharing with the other ARDs, such as cardiovascular diseases, type 2 diabetes, and late-onset cancer, the major risk factors and some basic mechanistic pillars that largely converge on inflammation (Franceschi et al., 2000; Leng and Edison, 2020; Sabbatinelli et al., 2021). Indeed, during aging a chronic, systemic, low-grade inflammation—named inflammaging—develops, representing a significant risk factor for both morbidity and mortality in elderly people (Franceschi et al., 2000). Accordingly, several pieces of evidence indicate that neuroinflammation plays a major role in the pathogenesis of AD, mainly through the activation of the innate immune system, including both central nervous system (CNS)-resident microglia and peripheral mononuclear phagocytes (Leng and Edison, 2020). Also, pro-inflammatory cytokines, such as interleukin-18 (IL-18), or a combination of interferon-gamma (IFN-γ) and tumor necrosis factor-alpha (TNF-α) impact the processing and production of the Aβ peptide (Blasko et al., 1999; Sutinen et al., 2012; Riphagen et al., 2020). Recent evidence suggested an association between the routine inflammatory biomarker C-reactive protein (CRP) and cognitive decline among a large racially diverse cohort of older adults (Arce Rentería et al., 2020).

Moreover, genome-wide association studies identified significant correlations between polymorphisms in genes participating in the innate immune response and the incidence of LOAD (Lambert et al., 2009; Jones et al., 2010; Jonsson et al., 2013). Given the lack of a proper understanding of the molecular mechanisms of AD development and progression and the difficulties of establishing an early diagnosis, effective treatments for AD are still not available. In this framework, the search for more effective biomarkers for monitoring cognitive impairment is imperative.

In recent years, microRNAs (miRNAs, miRs), endogenous short non-coding single-stranded RNA molecules, emerged as cost-effective biomarkers for the diagnosis of various human ARDs, including AD. Several studies identified specific miRNAs differentially expressed in postmortem brain tissues of AD patients (Clement et al., 2016; Henriques et al., 2020; Wingo et al., 2020). Since they can be also actively secreted by living cells in all body fluids as mediators of epigenetic information, miRNAs have been investigated also in cerebrospinal fluid (CSF; Marchegiani et al., 2019; Kumar and Reddy, 2021), as well as in plasma/serum of AD patients (Leidinger et al., 2013; Kumar et al., 2017; Mengel-From et al., 2018). Increasing evidence suggests that circulating miRNAs loaded onto different cargoes, such as proteins, lipoproteins, or vesicles (exosomes or microvesicles) could be able to cross the blood-brain barrier and target recipient cells (Blandford et al., 2018; Saeedi et al., 2019). Thus, it is conceivable that circulating miRNAs released by distal cells can reach the brain, and that miRNAs synthesized and released by CNS cells can be detectable in the bloodstream. However, one of the main challenges for the management of patients with ARDs is the identification of minimally invasive biomarkers of disease development and progression. Samples of brain tissue and CSF cannot be obtained from large cohorts of patients, especially from the oldest-old patients and from patients with mild cognitive impairment. Therefore, the evaluation of the clinical relevance of circulating miRNAs in AD patients is currently under increasing investigation.

A relatively small number of miRNAs involved in the regulation of inflammatory processes were previously identified as inflamma-miRNAs (Olivieri et al., 2013b), due to their ability to target key molecules belonging to the nuclear factor-κB (NF-κB) inflammatory pathway. NF-κB is considered as the master regulator of the pro-inflammatory programs activated by a plethora of triggers including cellular senescence (Hernandez-Segura et al., 2018), and its signaling has been extensively implicated in the development of the major ARDs, including AD (Jones and Kounatidis, 2017). Based on the evidence that progressive up-regulation of inflammatory genes and high levels of inflammatory signaling facilitates ARD development (Olivieri et al., 2013a; Xia et al., 2019), it is conceivable that circulating inflamma-miRNAs may be involved in the modulation or promotion of pathogenic signaling throughout the peripheral circulation and the CNS in AD. This hypothesis is supported by the evidence that some inflamma-miRNAs are also strongly expressed in human CSF fluid and brain tissue-derived extracellular fluid from AD patients (Olivieri et al., 2013a; Marchegiani et al., 2019).

We selected four inflamma-miRNAs—miR-17-5p, miR-21-5p, miR-146a-5p, and miR-126-3p—that are significantly expressed in AD brain tissues and are involved in the modulation of neuroinflammation targeting molecules of the NF-κB pathway (Sonntag et al., 2012; Ksiazek-Winiarek et al., 2013; Slota and Booth, 2019; Zhao et al., 2020).

The overall aim of our current investigation was to evaluate the diagnostic performance of these four inflamma-miRNAs and their potential relevance as biomarkers of AD-associated cognitive impairment.

## Materials and Methods

### Study Participants

One-hundred and sixteen patients with sporadic AD (age range: 65–89 years; mean age 77.2 ± 5.4 years; 36 men and 80 women) and 40 non-demented healthy age- (*p* = 0.10) and gender- (*p* = 0.51) matched control subjects (age range: 65–92 years; mean age 75.1 ± 7.5 years, 15 men and 25 women) were enrolled for the study. Patients with clinically probable sporadic AD were enrolled within the framework of the Italian National Cronos Project (CP), involving the AD Evaluation Unit (UVA) of IRCCS INRCA, Ancona. Clinical diagnosis of AD was based upon the Diagnostic and Statistical Manual of Mental Disorders (DMS IV-R) and the National Institute of Neurological Communicative Disorders and Stroke–Alzheimer’s Disease and Related Disorders Association (NINCDS–ADRDA) criteria (McKhann et al., 1984). AD patients with diabetes, severe cardiovascular comorbidities, chronic inflammatory disorders, or cancer were excluded. The status of the ApoE ε4 genotype was previously assessed (Lescai et al., 2011). Healthy subjects were selected from a larger population of ambulatory subjects attending the facilities of IRCCS INRCA for routine blood examinations. Information on their healthy state was assessed by questionnaires, laboratory assays, and physical examination (Olivieri et al., 2009). Subjects were considered healthy if, at the time of blood collection, they were free of clinically evident major diseases. Subjects with a Cumulative Illness Rating Scale (*CIRS*) >2, which indicates a comorbid state, were excluded. Determination of high-sensitivity C-reactive protein (hsCRP) was conducted on a Cobas 6000 automated analyzer (Roche Diagnostic, Meylan, France) using an immunoturbidimetric method. Then, the whole population was stratified according to the Mini-Mental State Examination (MMSE) score into cognitively normal subjects (MMSE ≥25), and subjects with mild (17 < *MMSE* < 25) or severe (MMSE≤17) cognitive impairment. The study protocols were approved by the Institutional Review Board of IRCCS INRCA, Ancona, Italy. Informed consent was obtained from healthy subjects and a relative of each AD patient.

### Sample Collection, RNA Isolation and Quantitation

Peripheral venous blood samples from the subjects analyzed were processed within 2 h from the collection by centrifugation into EDTA-coated tubes at 2,000 *g* at 4°C for 20 min. Plasma samples were then stored at −80°C until further analyzed.

Total RNA was extracted from 100 μl of plasma-EDTA using the total RNA Purification kit from Norgen Biotek Corporation (Thorold, ON, Canada) according to the manufacturer’s guidelines. Circulating miRNA levels were analyzed as described in Olivieri et al. (2014) using a modified real-time approach with the TaqMan miRNA reverse transcription kit and the miRNA assay from Applied Biosystems (Foster City, CA, USA). qPCR reactions were performed on a RotorGene Q HRM instrument (Qiagen, Germany). The plasma levels of circulating miRNAs are reported as relative expression (RE) normalized to the mean of spiked-in synthetic non-human cel-miR-39. The relative expression of each miRNA was reported as 2^−ΔCt^, with ΔCt being the difference between the Cts of a specific miRNA and those of the cel-miR-39. Cel-miR-39 was used also for the assessment of miRNA recovery from biological samples. Only those samples with a cel-miR-39 recovery higher than 95% were processed for inflamma-miRNA quantification. Each reaction was performed in duplicate.

### Statistical Analysis

Continuous data were tested for normality by Shapiro–Wilk’s test and are presented as mean ± standard deviation (SD) or median (IQR) for normally and non-normally distributed variables, respectively. Categorical variables are reported as relative frequencies and compared using Chi-squared tests. Mann–Whitney *U*-test was used for pair-wise comparisons between healthy controls and AD patients. Comparison among three groups was performed with the Kruskal–Wallis test. The relationships between the levels of each miRNA were calculated by Spearman rank correlation test and regression analysis. Receiver operating characteristics (ROC) curves were generated and the area under the curve (AUC) was used to check the diagnostic accuracy of miRNAs in AD and MMSE decline. Youden’s method was used to compute the optimal cut-off values (Youden, 1950). Data analysis was carried out with the IBM SPSS Statistics 26.0 for Windows software (SPSS Incorporation, Chicago, IL, USA). Statistical significance was defined as a two-tailed *p-value* < 0.05.

## Results

**Figure 1:**
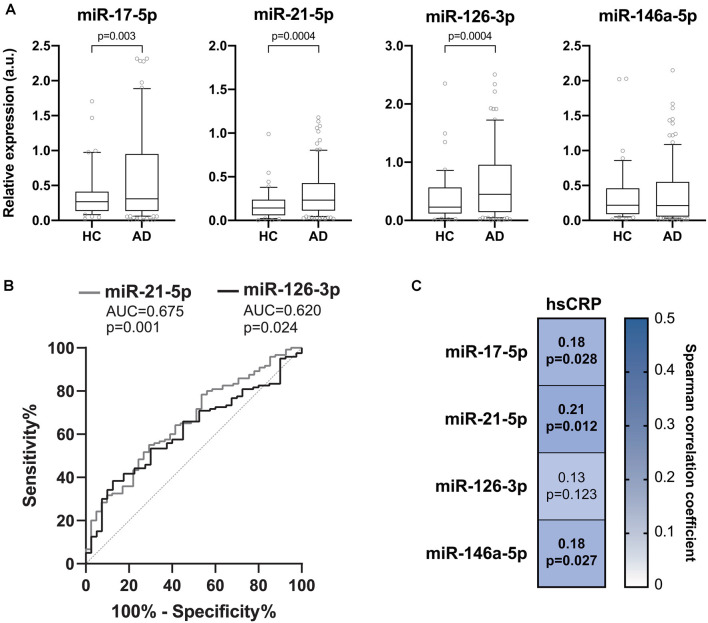
Inflamma-miR levels in healthy and Alzheimer’s disease (AD) subjects. **(A)** Boxplots showing the plasma miR-17-5p, miR-21-5p, miR-126-3p and miR-146a-5p levels in healthy control (HC, *n* = 40) and in Alzheimer’s disease (AD, *n* = 116) subjects. Data are presented as arbitrary units (a.u.) of selected miRNA relative expression (RE) normalized with cel-miR-39. *P*-values for Mann-Whitney *U* tests. **(B)** Receiver-operating characteristic (ROC) curves of miR-21-5p and miR-126-3p RE (a.u.) for the diagnosis of AD. The area under the curves (AUCs) and corresponding *p*-values are reported. **(C)** Correlation plot showing Spearman’s correlations between circulating levels of inflamma-miRs and serum hsCRP. Spearman’s rho coefficients and *p-*values are reported. The background color depends on the magnitude of the correlation. Significant correlations are marked in bold.

The expression levels of the inflamma-miRs miR-17-5p, -21-5p, -126-3p, and -146a-5p were analyzed in the plasma of 116 AD patients and compared to those obtained in healthy age- and gender-matched subjects (healthy control group, HC). Clinical characteristics of the enrolled subjects are listed in Table 1. The relative expressions of the four miRNAs showed a high degree of reciprocal correlation, with Spearman’s rho coefficients ranging from 0.571 to 0.730 (*p* < 0.001 for all miRNA pairs). The relative expressions of the four miRNAs are reported in Figure 1A. MiR-17-5p, miR-126-3p, and miR-21-5p were significantly up-regulated in AD patients compared to HC. On the contrary, miR-146a-5p did not show any significant difference between patients and controls (Figure 1A). To evaluate the ability of the selected inflamma-miRs to discriminate between HC and AD patients, we performed ROC curve analysis, unraveling significant AUCs for miR-21-5p (*AUC* = 0.675, *p* = 0.001) and miR-126-3p (*AUC* = 0.620, *p* = 0.024; Figure 1B). The optimal relative expression cut-off for miR-21-5p (0.214) was associated with a sensitivity of 55.0%, a specificity of 70.7%, a positive predictive value (PPV) of 84.6%, and a negative predictive value (NPV) of 34.9%. For miR-126-3p, the optimal cut-off (0.653) was associated with a sensitivity of 38.3%, a specificity of 87.5%, a PPV of 90.2%, and a NPV of 32.2%. We then assessed the correlation between plasma hsCRP and the circulating levels of inflamma-miRs in the whole cohort. Significant Spearman’s positive correlations were observed for miR-17-5p (*p* = 0.028), miR-21-5p (*p* = 0.012), and miR-146a-5p (*p* = 0.027; Figure 1C).

**Table 1:** Clinical and demographic characteristics of healthy control subjects (HC) and patients with Alzheimer’s disease (AD).

Variable	HC (*n* = 41)	AD (*n* = 116)	*p*-value
Age (years)	75.1 (7.5)	77.2 (5.4)	0.097
Gender (Males, %)	15 (37%)	36 (31%)	0.514
MMSE (score)	28.5 (2.3)	19.4 (3.9)	<0.001
APOE ε4 allele (*n*, %)	7 (17%)	55 (47%)	<0.001
hsCRP (mg/L)	0.48 (1.61)	0.65 (1.56)	0.541
Time from AD diagnosis (years)	-	3.4 (1.3)	-
ADL (score)	-	5.0 (1.4)	-
IADL (score)	-	4.0 (2.2)	-
ADAS-Cog (score)	-	20.1 (17.1)	-
Treatments (*n*, %)			
Donepezil	-	70 (60%)	-
Rivastigmine	-	39 (34%)	-
Galantamine	-	14 (12%)	-
Antidepressants	2 (5%)	44 (38%)	<0.001
Antipsychotic drugs	0 (0%)	40 (34%)	-
Anxiolytics/sedatives/hypnotics	6 (15%)	75 (65%)	<0.001
Antihypertensive drugs	20 (49%)	72 (62%)	0.138
Antiplatelet drugs	9 (22%)	63 (54%)	<0.001

*ADAS-Cog, Alzheimer’s Disease Assessment Scale-Cognitive Subscale; ADL, activities of daily living; hsCRP, high-sensitivity C-reactive protein; IADL, instrumental activities of daily living; MMSE, Mini-Mental State Examination. Data are expressed as mean (standard deviation) or number of subjects (percentage). *P*-value from *t*-test for continuous variables and chi-squared tests of association for categorical variables*.

**Figure 2:**
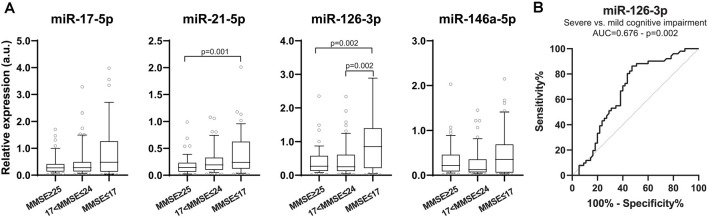
Inflamma-miR levels in subjects are grouped according to Mini-Mental State Examination (MMSE) score. **(A)** Boxplots showing plasma miR-17-5p, miR-21-5p, miR-126-3p and miR-146a-5p levels in subjects with normal cognition (MMSE ≥ 25, *n* = 50), mild (18 ≤ MMSE ≤ 24, *n* = 52) and severe (MMSE ≤ 17, *n* = 55) cognitive impairment evaluated through MMSE score. Data are presented as a.u. of selected miRNA RE normalized with cel-miR-39. *P*-values for Kruskal–Wallis test with *post hoc* Dunn test. **(B)** ROC curve of miR-126-3p RE (a.u.) for discriminating between mild and severe cognitive impairment. AUC and corresponding *p*-value are reported.

To evaluate whether the profiling of these circulating inflamma-miRNAs is AD stage-dependent, we grouped subjects according to their MMSE score in cognitively normal subjects (MMSE ≥25), and subjects with mild (17 < *MMSE* < 25) or severe (MMSE≤17) cognitive impairment. Interestingly, the expression levels of miR-21-5p and miR-126-3p were inversely correlated with MMSE scores (Figure 2A). Notably, miR-126-3p levels were significantly higher in AD patients with severe cognitive impairment compared with cognitively normal subjects (*p* = 0.002) and patients with mild cognitive impairment (*p* = 0.002), thus highlighting a progressive increase of miR-126-3p expression with worsening dementia. ROC curve analysis revealed a significant AUC only for miR-126-3p (*AUC* = 0.676, *p* = 0.002; Figure 2B), with an optimal cut-off (0.838) associated with a sensitivity of 52.7%, a specificity of 39.0%, a PPV of 80.6%, and a NPV of 62.9%.

## Discussion

The identification of minimally invasive biomarkers with diagnostic and/or prognostic value for AD is an urgent need, due to the strikingly high prevalence of AD in the population over 65 years. Here, we showed that the circulating levels of four selected inflamma-miRs, such as miR-17-5p, miR-126-3p, and miR-21-5p were significantly increased in AD patients compared to normal age-matched controls.

MiR-126-3p was first identified as an “endothelial miR,” given its ability to promote endothelial integrity maintenance and blood vessel formation (Zhong et al., 2018). Intriguingly, miR-126-3p modulates inflammation and innate immune response by targeting components of the NF-κB pathway and endothelial adhesion molecules, such as VCAM-1 (Harris et al., 2008). MiR-126-3p has been shown to affect the expression of EGFL7 (Nikolic et al., 2010), a secretory protein regulating angiogenesis that is involved also in adult neurogenesis as a repair mechanism in neurodegenerative disorders (Bicker et al., 2017). In the CNS, miR-126-3p is involved in regulating the insulin/IGF pathway as well as in modulating neuronal vulnerability to the toxic insult (Kim et al., 2016). Indeed, elevated miR-126 levels were proven to increase Aβ42 toxicity and to interfere with the neuroprotective effect of IGF-1 by downregulating the expression of members of the PI3K and ERK pathways (Kim et al., 2016). On the other hand, the role of miR-126 in the maintenance of vascular integrity has been recapitulated also in a model of intracerebral hemorrhage, where it was shown to improve the blood-brain barrier stability and to promote favorable cognitive outcomes following the acute event (Xi et al., 2017). Collectively, this evidence suggests that miR-126 may provide an important mechanistic link between vascular dysfunction and neurotoxicity in the pathogenesis of neurodegenerative diseases, including AD.

Also, miR-17-5p has been initially described as a senescence-associated miRNA with a role in the modulation of innate immunity (Zhu et al., 2013). It has been shown that miR-17-5p down-regulate amyloid precursor protein (APP) expression *in vitro* and *in vivo* (Delay et al., 2011). The increased expression of circulating miR-17-5p that we observed in AD patients compared to healthy control subjects could be considered as a compensatory mechanism to restrain the increased APP synthesis and accumulation observed in AD patients.

MiR-21-5p is largely studied in the context of inflammation and aging. Since miR-21-5p is down-regulated in healthy elderly subjects and up-regulated in patients affected by many age-associated disorders, circulating miR-21-5p levels represent one of the best candidates to track the aging trajectories (Sonntag et al., 2012). Here, we show a significant miR-21-5p increase in AD patients with severe cognitive impairment, thus supporting our hypothesis. Interestingly, miR-21-5p was shown to be enriched in brain extracellular vesicles in an animal model of traumatic brain injury (Harrison et al., 2016). Moreover, miR-21 has been proposed as a component of a neuronal stress response in many models of neurodegeneration and neuroinflammation (Buller et al., 2010; Shi et al., 2012; Zhang et al., 2012; Ge et al., 2014), including AD (Hwang et al., 2019). Overall, increasing evidence supports a neuroprotective role for miR-21 in the CNS (Buller et al., 2010), albeit its precise role in modulating neuroinflammation has still to be clarified. Conflicting reports are available on circulating miR-21 levels in patients with AD (Burgos et al., 2014; Gámez-Valero et al., 2019), probably due to the great variability in the clinical characteristics of patients enrolled in the different studies (disease stages and time from diagnosis), and the heterogeneity of analyzed biological samples (plasma, serum, and CSF).

Surprisingly, circulating levels of miR-146a-5p are not significantly different between AD patients and age-matched healthy control subjects. However, our results are as per a previous report (Maffioletti et al., 2019).

As previously reported by our research group (Olivieri et al., 2012, 2014; Mensà et al., 2019), the ability of inflamma-miRNAs to modulate the low-grade systemic-inflammation could be relevant for a healthy aging trajectory (Franceschi et al., 2000). Notably, we observed significant positive correlations between the levels of the systemic inflammatory marker hsCRP and three of the evaluated circulating inflamma-miRs, i.e., miR-17-5p, -21-5p, and -146a-5p, reinforcing their role as mediators of the chronic inflammatory status that accompanies human aging (Olivieri et al., 2012; Wade et al., 2020).

Increasing experimental data suggest that aging is the major risk factor for the most common ARDs. Assuming that aging and ARDs share a common set of basic biological mechanisms, they can be considered as parts of a continuum where precise boundaries do not exist (Franceschi et al., 2018). Therefore, if ARDs, including AD, are manifestations of accelerated aging, it is urgent to identify markers capable of distinguishing between healthy and unhealthy aging. In this framework, we proposed that circulating inflamma-miRNAs could represent valuable tools to detect early deviations from a healthy aging trajectory, rather than biomarkers of specific ARDs (Olivieri et al., 2017).

Overall, our results support the hypothesis that circulating inflamma-miRs, especially miR-17-5p, -21-5p, and -126-3p, could represent minimally invasive biomarkers for AD diagnosis and progression. Importantly, miR-21-5p and miR-126-3p provide additional information on the extent of AD-associated cognitive impairment. Since the mechanisms and risk factors associated with the decline of cognitive function in AD are still elusive, comparison of patients with mild degrees of dementia with severe and normal counterparts could be relevant from a diagnostic and/or prognostic point of view. In this regard, it is worth noting that our study indicates that plasma levels of miR-126-3p showed a statistically significant relationship with cognitive impairment, assessed by the means of declining MMSE. In particular, miR-126-3p showed a good discriminatory ability between mild and severe cognitive impairment, even if the ability to stratify the mild cognitive impairment in comparison with the normal condition was not highlighted.

Here, we provided further evidence that the combination of circulating inflamma-miR analysis with the already established diagnostic methods could confer important advantages both in AD diagnosis and in the evaluation of treatment efficacy. Further investigations are required to confirm our results in multicentric larger cohorts.

## Data Availability Statement

The raw data supporting the conclusions of this article will be made available by the authors, without undue reservation.

## Ethics Statement

The studies involving human participants were reviewed and approved by IRCCS INRCA, Ancona, Italy. The patients/participants provided their written informed consent to participate in this study.

## Author Contributions

SG, GS, SA, ML, MG, and GM performed real-time PCR analyses. TR, GPe, and AB enrolled patients and control subjects and collected blood samples. RG and GPi performed hsPCR measurements. RG, AP, and MA reviewed the article considering their experience in the field. AG and JS performed the statistical analysis, participated in the drafting of the article, and prepared the figures. FO and FF conceived the study and drafted the article. All authors contributed to the article and approved the submitted version.

## Conflict of Interest

The authors declare that the research was conducted in the absence of any commercial or financial relationships that could be construed as a potential conflict of interest.
